# Distorted zinc coordination polyhedra in bis­(1-eth­oxy-2-{[(2-meth­oxy­eth­yl)imino]­meth­yl}propan-1-olato)zinc, a possible CVD precursor for zinc oxide thin films

**DOI:** 10.1107/S2056989022001475

**Published:** 2022-02-10

**Authors:** Keneshia O. Johnson, Antionette Brown, Gabriella Farris, Alexabria Starks, Ray J. Butcher, Jason S. Matthews

**Affiliations:** a Department of Physics, Chemistry and Mathematics, Alabama A & M University, 4900 Meridian Street N, Huntsville, Alabama 35811-7500, USA; b Department of Chemistry, Howard University, 525 College Street NW, Washington DC 20059, USA

**Keywords:** crystal structure, β-imino­esterate complex, ketoiminato zinc complex

## Abstract

A new metal–organic precursor for the chemical vapor deposition of zinc oxide thin films, [Zn(C_9_H_16_NO_3_)_2_], has been synthesized and characterized by ^1^H and ^13^C NMR spectroscopy, single-crystal X-ray diffraction and thermogravimetric analysis. The asymmetric unit of the title compound consists of two mol­ecules (*Z*′ = 2), with different zinc coordination polyhedra. In one mol­ecule, the metal atom is in a distorted trigonal–bipyramidal ZnN_2_O_3_ environment (τ_5_ = 0.192) with a long bond to an ether O donor atom [Zn—O = 2.727 (6) Å]. In the other, the Zn atom is in a distorted ZnN_2_O_4_ octa­hedral environment with long bonds to the ether O donors of both ligands [Zn—O = 2.514 (4) and 2.661 (4) Å; O—Zn—O = 82.46 (14)°]. The crystal structure features weak C—H⋯·O inter­actions.

## Abstract 2

The coordination polyhedra of the zinc atoms in the title complex display long Zn—O bonds as parts of distorted trigonal bipyramids and octa­hedra.

## Chemical context

Zinc oxide is of considerable current inter­est in materials science because it is a semiconductor with a band gap of 3.37 eV and it possesses high electron mobility, a high exciton binding energy of 60 meV, strong room-temperature luminescence, photoelectric response, high transparency, and high photocatalytic activity (Ganesh *et al.*, 2017 ▸; Das & Sarkar, 2017 ▸). As a result of these favorable properties, ZnO has potential applications in solar cells, sensors, ultra-violet laser diodes, actuators, field-emission devices, photocatalysts and piezoelectric devices (Galstyann *et al.*, 2015 ▸; Hong *et al.*, 2017 ▸). The identification of a viable technique that is capable of depositing zinc oxide thin films of high purity and high quality is a significant challenge. Metal–organic chemical vapor deposition (MOCVD) has proven to be a promising method for depositing high-quality ZnO thin films at a high growth rate over a large area (Malandrino *et al.*, 2005 ▸). The success of the MOCVD process depends heavily on the precursor. An ‘ideal’ MOCVD precursor should be volatile, exhibit a sufficiently large temperature window between evaporation and film deposition, and decompose without the incorporation of residual impurities. Diethyl zinc, Zn(C_2_H_5_)_2_, in combination with an oxygen source, H_2_O, or *R*OH is the traditional precursor for depositing ZnO thin films (Smith, 1983 ▸). As a result of the pyrophoric nature of the alkyl zinc reagents and the gas-phase pre-reaction that results in precursor decomposition and film contamination, alternative precursors such as alkoxide, dialkyl zinc precursors of acetate and acetyl­acetonate have been employed (Sato *et al.*, 1994 ▸). The drawback with these precursors is that impurities are often incorporated in the deposited ZnO films. These disadvantages have resulted in a search for single-source precursors. A single-source precursor is one that has the oxygen already present in the precursor, thereby eliminating the need for an external oxygen source. 
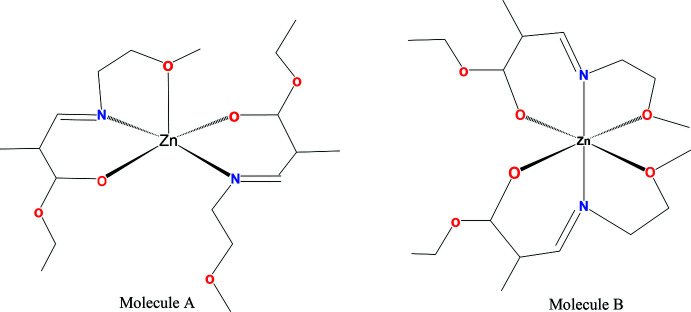


The synthesis of two thermally stable ketoiminato zinc complexes [Zn{[(CH_2_)_*x*_OCH_3_]NC(CH_3_)=C(H)C(CH_3_)=O}_2_] (**1**: *x* = 2; **2**: *x* = 3) were reported with melting points as low as 330 K (Barreca *et al.*, 2010 ▸; Bekermann *et al.*, 2010*a* ▸,*b* ▸). In another case, ketoiminato zinc complexes that incorporate ether O-donor atoms have shown promise (Cosham *et al.*, 2015 ▸). With these favorable results in mind, we decided to further explore the β-enamino­alk­oxy­ester ligand platform. Our research group has demonstrated that high-quality ZnO thin films with fewer impurities can be accomplished by utilizing Zn–bis-β-imino­esterate complexes (Matthews *et al.*, 2006 ▸; Onakoya *et al.*, 2011 ▸; Gbemigun *et al.*, 2019 ▸). Studies have shown that the organic ligand attached to the N moiety of the zinc complex has a significant effect on the level of carbon incorporated into the deposited ZnO thin film (Manzi *et al.*, 2015 ▸), thus the investigation of such compounds with different substituents at the N atom is of significant inter­est in improving precursors for these ZnO films. Herein, the synthesis, characterization and crystal structure of the title compound **1** are reported.

## Structural commentary

The synthesis of [Zn(C_9_H_16_NO_3_)_2_] (**1**), was carried out by the direct reaction of **1a** with diethyl zinc in a 2:1 molar ratio under an inert atmosphere of nitro­gen utilizing Schlenk techniques to afford white single crystals of complex **1**. The ^1^H-NMR and ^13^C-NMR spectra of **1** contain the characteristic resonances in the expected regions. The ^1^H-NMR spectrum in particular shows the absence of the N—H resonance (δ = 8.63) that was present in the free ligand (**1a**), indicating the absence of any starting material. Generally, the introduction of a Lewis acidic metal center into the ligand sphere results in the proton and carbon resonances being shifted downfield (Matthews *et al.*, 2006 ▸). This was not observed in this study: in going from the free ligand (**1a**) to complex **1** most of the proton and carbon resonances were slightly shifted upfield. This inconsistency suggests that the electron density in the chelate ring of **1** is not completely delocalized around the ring. If complete delocalization was observed, the carbon atoms and protons in the complex would have been deshielded and the resonances would have been shifted downfield.

**Figure 1:**
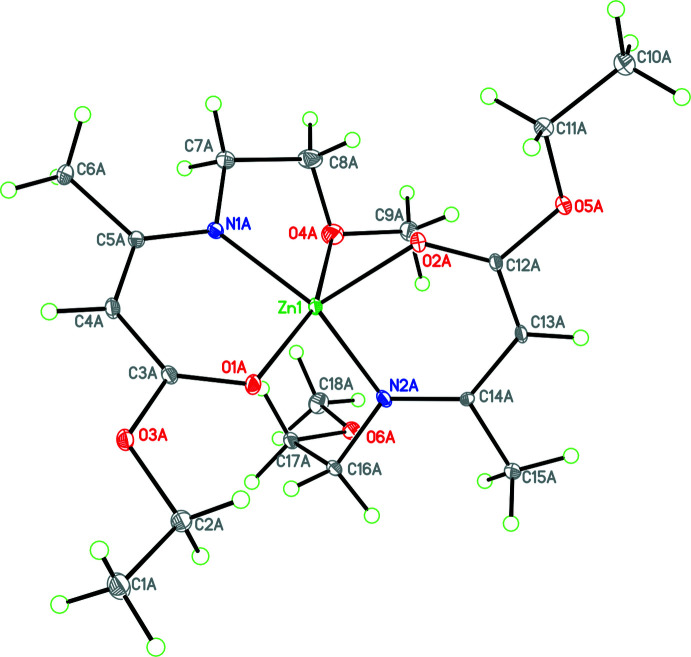
The mol­ecular structure of mol­ecule *A* showing the long Zn—O (ether) inter­action influencing the conformation of the substituent. Atomic displacement parameters are shown at the 30% probability level.

The title complex, C_18_H_32_N_2_O_6_Zn, **1**, crystallizes in the monoclinic space group *P*2_1_/*c* with eight mol­ecules in the unit cell, thus two in the asymmetric unit (*Z*′ = 2 and named as *A* and *B* for the purposes of discussion**)**, which have adopted different metal-ion coordinations and conformations (Table 1 ▸). In mol­ecule *A* (Fig. 1 ▸), the Zn atom is in a distorted trigonal–bipyramidal ZnN_2_O_3_ environment (τ_5_ = 0.192; Addison *et al.*, 1984 ▸) with a long bond to an ether O donor atom [Zn1—O4*A* = 2.727 (6) Å] and the ligand N atoms in the axial sites [N1*A*—Zn1—N2*A* = 144.05 (19)°].

**Figure 2:**
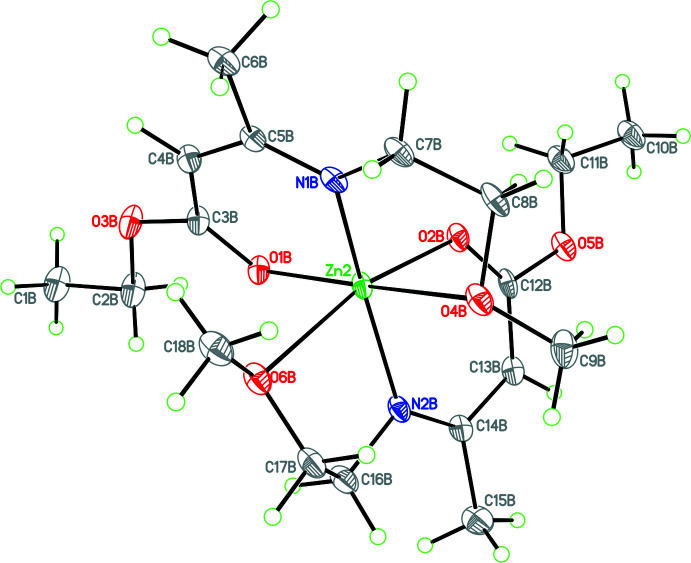
The mol­ecular structure of mol­ecule *B* (major disorder component only) showing long Zn—O (ether) bonds (arranged in a *cis* fashion) resulting a distorted octa­hedral coordination for the metal atom. Atomic displacement parameters are shown at the 30% probability level.

In mol­ecule *B* (Fig. 2 ▸), the Zn atom is in a distorted octa­hedral environment with long bonds to the ether O donors of both ligands [Zn—O bond lengths of 2.514 (4) and 2.661 (4) Å; O6*B*—Zn2—O4*B* bond angle = 82.46 (14)°]. Also in *B* there is disorder in some of the ethyl substituent groups [occupancies of 0.717 (13)/0.283 (13) and 0.68 (3)/0.32 (3)]. In *B*, the ether donor atoms are arranged in a *cis* fashion so the complex does not exhibit tetra­gonal distortion. There are significant differences in the short Zn—O and Zn—N bond lengths in the two mol­ecules [Zn—O = 1.974 (4)/2.025 (4) and 2.017 (4)/2.045 (4) Å: Zn—N = 1.958 (4)/1.966 (5) and 1.990 (4)/2.004 (5) Å for *A* and *B*, respectively].

Both keto­imine chelate rings are almost planar (r.m.s. deviations of 0.018 and 0.026 Å for mol­ecule *A* and 0.002 and 0.014 Å for mol­ecule *B*) with the zinc atoms deviating from the respective planes by 0.089 (6)/0.220 (6) Å and 0.248 (2)/0.030 (7) Å for *A* and *B*, respectively. The dihedral angles between the chelate planes in **1** are 71.4 (1) and 77.3 (1)° for the *A* and *B* mol­ecules, respectively.

## Supra­molecular features

As far as the packing of **1** is concerned, there are both inter- and intra­molecular C—H⋯O inter­actions (Table 2 ▸). While these are presumably weak based on their length, it can be seen that the intra­molecular C—H⋯O inter­actions influence the conformations adopted by the side chains for both mol­ecules (see Figs. 1 ▸, 2 ▸ and 3 ▸).

## Database survey

Four closely related structures to **1** have been reported [Cambridge Structural Database (Groom *et al.*, 2016 ▸) refcodes SUPXEI, SUPXIM, SUPXOS and SUPXUY; Cosham *et al.*, 2015 ▸], which incorporate both a keto­imine ligand along with ether O donors. In each case the ether donors are in *cis* positions with Zn—O bond lengths ranging from 2.316 to 2.575 Å.

There are five previously reported structures of ketoiminato zinc complexes (EFIWEY and EFIWIC, Gbemigun *et al.*, 2019 ▸; IDAWAN, Onakoya *et al.*, 2011 ▸; WELSOW, Matthews *et al.*, 2006 ▸; YUJMAT, Manzi *et al.*, 2015 ▸). These all contain zinc in a slightly distorted tetra­hedral environment [τ_4_′ = 0.65, 0.65 0.73, 0.82, 0.79 and 0.73 (Okuniewski *et al.*, 2015 ▸), respectively, for EFIWEY, EFIWIC, IDAWAN, WELSOW and YUJMAT]. However EFIWEY and EFIWIC are both dimers with only one imino­esterate ligand attached to each zinc atom so IDAWAN, WELSOW and YUJMAT are the most relevant structures to the present example.

The asymmetry in the out-of-plane deviations of the Zn atoms in **1** noted above is a pattern that is repeated in the three most closely related structures (deviations = 0.084/0.341, 0.146/0.373 and 0.152/0.208 Å for IDAWAN, WELSOW and YUJMAT, respectively). The dihedral angles between the chelate-ring planes for IDAWAN, WELSOW and YUJMAT are 89.29, 81.0 and 72.47°, respectively.

## Experimental

All chemicals were purchased from Aldrich and used without further purification. The ^1^H and ^13^C-NMR spectra were recorded with a Bruker AVANCE 400MHz Ultra Shield^TM^ NMR spectrometer. Chemical shifts for ^1^H (400MHz) and ^13^C (100MHz) were referenced to CDCl_3_ and reported in ppm. Thermogravimetric analyses were performed under a nitro­gen atmosphere at 1atm using a Perkin–Elmer thermogravimetric analyzer series 7 at a heating rate of 10°C min^−1^. All manipulations were carried out using oven dried, standard reflux glassware consisting of a condenser connected to a round-bottom flask. Distillation was performed using oven-dried micro-still apparatus.

### Synthesis and crystallization

**Synthesis of ethyl-3-** ***N*** **-(2-meth­oxy­ethyl­amino)­but-2-enoate (1a)**

Ethyl aceto­acetate (5.00 g, 38.42 mmol) and 2-meth­oxy­ethyl­amine (5.77 g, 76.84 mmol) were added to a 100 ml round-bottom flask *via* syringe. The solution was refluxed for 1h with constant stirring. The resulting mixture was allowed to cool to room temperature and approximately 30 ml of hexane was added to dissolve the product. The solution was then dried over anhydrous sodium sulfate. The resulting mixture was then filtered, and the solvent was evaporated *in vacuo* to afford a viscous yellow oil. This crude product was then purified *via* vacuum distillation to afford a viscous light-yellow oil (**1a**) (yield 73.02%, 5.22 g), b.p. 389–396 K at 1.2 mm Hg; ^1^H NMR 400 MHz, CDCl_3_, δ ppm: 1.21 (*t*, 3H, (OCH_2_CH_3_), 1.90 (*s*, 3H, CH_3_CN), 3.35 (*s*, 3H, OCH_3_), 3.36 (*q*, 2H, NCH_2_CH_2_), 3.46 (*t*, 2H, OCH_2_CH_2_), 4.05 (*q*, 2H, OCH_2_CH_3_), 4.43 (*s*, 1H, CCHCO), 8.63 (*br s*, 1H, NH); ^13^C NMR 100 MHz, CDCl_3_, δ ppm: 14.57 [OCH_2_CH_3_], 19.43 [CH_3_CN], 42.78 [CH_2_CH_2_N], 58.20 [OCH_3_], 58.99 [OCH_2_CH_3_], 71.80 [OCH_2_CH_2_], 82.60 [CCHCO], 161.55 [CH_3_CN], 170.44 [CHCO].

**Synthesis and crystallization of [Zn (C_9_H_16_NO_3_)_2_] (1)**

50ml of dried hexa­nes, ethyl-3-*N*-(2-meth­oxy­ethyl­amino) butano­ate **(1a)** (6.87 g, 36.5 mmol) and a stir bar were added to a 250 ml Schlenk flask under an inert atmosphere of nitro­gen. The mixture was degassed with N_2_ gas for approximately fifteen minutes then diethyl zinc (2.25 g, 18.25 mmol) was added. The resulting mixture was refluxed for 4 h with constant stirring. The solvent was removed *in vacuo* at room temperature to afford a viscous yellow oil. The yellow oil was recrystallized from a solution in dry hexa­nes for 48 h at 243 K to afford white needle-like crystals. The hexa­nes were removed using a cannula and the white needle-like crystals were purified by washing with cold 10 ml portions of dried hexa­nes (yield 71.7%, 5.73 g), m.p. 311.0–311.2 K. ^1^H NMR 400 MHz, (CDCl_3_, ppm): δ 1.18 (*t*, 6H, (OCH_2_CH_3_), 1.92 (*s*, 6H, CH_3_CN), 3.20 (*s*, 6H, OCH_3_), 3.43 (*m*, 4H, NCH_2_CH_2_), 3.43 (*m*, 4H, OCH_2_CH_2_), 4.03 (*q*, 4H, OCH_2_CH_3_), 4.28 (*s*, 2H, CCHCO); ^13^C NMR 100 MHz, CDCl_3_, δ ppm: 15.01 [OCH_2_CH_3_], 22.87 [CH_3_CN], 49.66 [CH_2_CH_2_N], 58.87 [OCH_3_], 59.01 [OCH_2_CH_3_], 72.37 [OCH_2_CH_2_], 78.14 [CCHCO], 171.37 [CH_3_CN], 172.31 [CHCO].

### Refinement

Crystal data, data collection and structure refinement details are summarized in Table 3 ▸. This was a highly air-sensitive compound and the best available crystal was chosen. However it was non-merohedrally twinned with multiple components. Integration and refinement using the hklf5 (twinned) file was not successful so the hklf4 file was used. Consequently there are two significant difference peaks in chemically unreasonable positions. A face-indexed absorption correction was applied but there are still some residual peaks near the metal atoms. For one of the asymmetric mol­ecules there is disorder in some of the ethyl substituents. These were constrained to have similar metrical parameters and refined with occupancy factors of 0.717 (13)/0.283 (13) and 0.68 (3)/0.32 (3). A riding model was used for the H atoms with atomic displacement parameters = 1.2*U*_eq_(C) [1.5*U*_eq_(CH_3_)], with C—H bond lengths ranging from 0.95 to 0.99 Å.

## Supplementary Material

Crystal structure: contains datablock(s) I. DOI: 10.1107/S2056989022001475/hb8009sup1.cif

Structure factors: contains datablock(s) I. DOI: 10.1107/S2056989022001475/hb8009Isup2.hkl

CCDC reference: 2150564

Additional supporting information:  crystallographic information; 3D view; checkCIF report

## Figures and Tables

**Figure 3:**
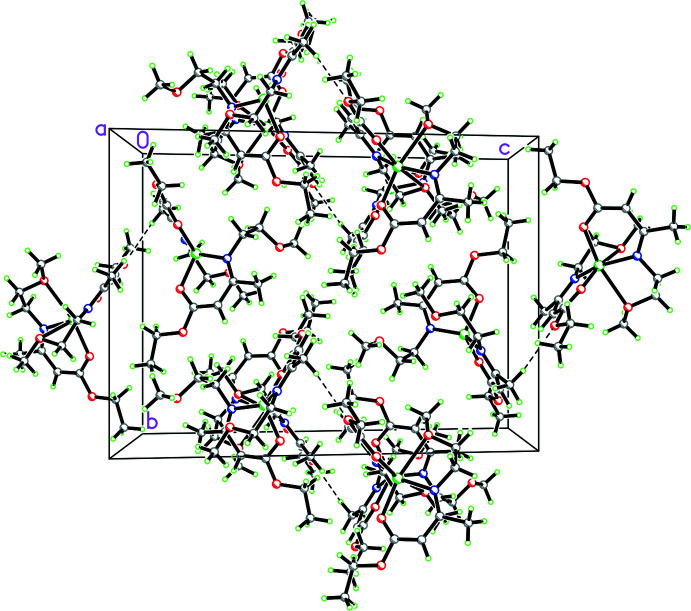
Packing diagram for **1** showing both the intra- and inter­molecular C—H⋯O inter­actions.

**Table 1:** Selected geometric parameters (Å, °)

Zn1—N1*A*	1.958 (4)	Zn2—N2*B*	2.004 (5)
Zn1—N2*A*	1.966 (5)	Zn2—O1*B*	2.017 (4)
Zn1—O2*A*	1.974 (4)	Zn2—O2*B*	2.045 (4)
Zn1—O1*A*	2.025 (4)	Zn2—O6*B*	2.514 (4)
Zn1—O4*A*	2.727 (6)	Zn2—O4*B*	2.661 (4)
Zn2—N1*B*	1.990 (4)		
			
N1*A*—Zn1—N2*A*	144.05 (19)	N1*B*—Zn2—O2*B*	101.90 (17)
N1*A*—Zn1—O2*A*	112.56 (17)	N2*B*—Zn2—O2*B*	92.60 (18)
N2*A*—Zn1—O2*A*	94.98 (17)	O1*B*—Zn2—O2*B*	102.98 (17)
N1*A*—Zn1—O1*A*	95.04 (17)	N1*B*—Zn2—O6*B*	86.02 (16)
N2*A*—Zn1—O1*A*	96.25 (17)	N2*B*—Zn2—O6*B*	76.39 (17)
O2*A*—Zn1—O1*A*	110.72 (18)	O1*B*—Zn2—O6*B*	85.76 (16)
N1*A*—Zn1—O4*A*	71.83 (18)	O2*B*—Zn2—O6*B*	167.48 (16)
N2*A*—Zn1—O4*A*	84.34 (17)	N1*B*—Zn2—O4*B*	73.58 (16)
O2*A*—Zn1—O4*A*	93.49 (18)	N2*B*—Zn2—O4*B*	83.20 (17)
O1*A*—Zn1—O4*A*	155.58 (17)	O1*B*—Zn2—O4*B*	164.14 (14)
N1*B*—Zn2—N2*B*	152.5 (2)	O2*B*—Zn2—O4*B*	90.42 (15)
N1*B*—Zn2—O1*B*	95.08 (17)	O6*B*—Zn2—O4*B*	82.46 (14)
N2*B*—Zn2—O1*B*	104.33 (18)		

**Table 2:** Hydrogen-bond geometry (Å, °)

*D*—H⋯*A*	*D*—H	H⋯*A*	*D*⋯*A*	*D*—H⋯*A*
C6*A*—H6*AB*⋯O3*B* ^i^	0.98	2.63	3.568 (8)	161
C15*A*—H15*A*⋯O6*A*	0.98	2.64	3.396 (7)	134
C15*A*—H15*C*⋯O3*A* ^ii^	0.98	2.65	3.369 (7)	131
C18*A*—H18*A*⋯O3*A* ^iii^	0.98	2.60	3.304 (8)	129
C8*B*—H8*BA*⋯O2*B*	0.99	2.60	3.279 (7)	126

Symmetry codes: (i) 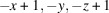 ; (ii) 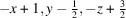 ; (iii) 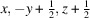 .

**Table 3:** Experimental details

Crystal data	
Chemical formula	[Zn(C_9_H_16_NO_3_)_2_]
*M*_r_	437.82
Crystal system, space group	Monoclinic, *P*2_1_/*c*
Temperature (K)	123
*a*, *b*, *c* (Å)	14.6212 (4), 14.8002 (4), 20.1288 (7)
β (°)	101.719 (3)
*V* (Å^3^)	4265.0 (2)
*Z*	8
Radiation type	Cu *K*α
μ (mm^−1^)	1.89
Crystal size (mm)	0.45 × 0.09 × 0.06

Data collection	
Diffractometer	Xcalibur, Ruby, Gemini
Absorption correction	Analytical (*CrysAlis PRO*; Rigaku OD, 2015 ▸)
*T*_min_, *T*_max_	0.484, 0.908
No. of measured, independent and observed [*I* > 2σ(*I*)] reflections	17610, 8591, 6698
*R*_int_	0.089
(sin θ/λ)_max_ (Å^−1^)	0.629

Refinement	
*R*[*F* ^2^ > 2σ(*F* ^2^)], *wR*(*F* ^2^), *S*	0.111, 0.277, 1.06
No. of reflections	8591
No. of parameters	537
No. of restraints	78
H-atom treatment	H-atom parameters constrained
Δρ_max_, Δρ_min_ (e Å^−3^)	4.24, −0.65

Computer programs: *CrysAlis PRO* (Rigaku OD, 2015 ▸), *SHELXS97* (Sheldrick, 2008 ▸), *SHELXL2018/3* (Sheldrick, 2015 ▸), *SHELXTL* (Sheldrick, 2008 ▸).

## supplementary crystallographic information

### Crystal data

**Table:**

[Zn(C_9_H_16_NO_3_)_2_]	*F*(000) = 1856
*M**_r_* = 437.82	*D*_x_ = 1.364 Mg m^−^^3^
Monoclinic, *P*2_1_/*c*	Cu *K*α radiation, λ = 1.54184 Å
*a* = 14.6212 (4) Å	Cell parameters from 6033 reflections
*b* = 14.8002 (4) Å	θ = 3.0–75.4°
*c* = 20.1288 (7) Å	µ = 1.89 mm^−^^1^
β = 101.719 (3)°	*T* = 123 K
*V* = 4265.0 (2) Å^3^	Needle, colorless
*Z* = 8	0.45 × 0.09 × 0.06 mm

### Data collection

**Table:**

Xcalibur, Ruby, Gemini diffractometer	6698 reflections with *I* > 2σ(*I*)
Detector resolution: 10.5081 pixels mm^-1^	*R*_int_ = 0.089
ω scans	θ_max_ = 76.0°, θ_min_ = 3.1°
Absorption correction: analytical (CrysalisPro; Rigaku OD, 2015)	*h* = −18→13
*T*_min_ = 0.484, *T*_max_ = 0.908	*k* = −18→17
17610 measured reflections	*l* = −25→24
8591 independent reflections	

### Refinement

**Table:**

Refinement on *F*^2^	Primary atom site location: structure-invariant direct methods
Least-squares matrix: full	Secondary atom site location: difference Fourier map
*R*[*F*^2^ > 2σ(*F*^2^)] = 0.111	Hydrogen site location: mixed
*wR*(*F*^2^) = 0.277	H-atom parameters constrained
*S* = 1.06	*w* = 1/[σ^2^(*F*_o_^2^) + (0.1511*P*)^2^ + 16.9004*P*] where *P* = (*F*_o_^2^ + 2*F*_c_^2^)/3
8591 reflections	(Δ/σ)_max_ = 0.001
537 parameters	Δρ_max_ = 4.24 e Å^−^^3^
78 restraints	Δρ_min_ = −0.65 e Å^−^^3^

### Special details

**Table:**

Geometry. All esds (except the esd in the dihedral angle between two l.s. planes) are estimated using the full covariance matrix. The cell esds are taken into account individually in the estimation of esds in distances, angles and torsion angles; correlations between esds in cell parameters are only used when they are defined by crystal symmetry. An approximate (isotropic) treatment of cell esds is used for estimating esds involving l.s. planes.

### Fractional atomic coordinates and isotropic or equivalent isotropic displacement parameters (Å^2^)

**Table:**

	*x*	*y*	*z*	*U*_iso_*/*U*_eq_	Occ. (<1)
Zn1	0.35835 (5)	0.13597 (4)	0.66026 (4)	0.0310 (2)	
O1A	0.4279 (3)	0.2317 (3)	0.6185 (2)	0.0357 (9)	
O2A	0.3745 (3)	0.0160 (3)	0.6213 (2)	0.0387 (9)	
O3A	0.4400 (3)	0.3643 (3)	0.5675 (2)	0.0378 (9)	
O4A	0.2400 (4)	0.0727 (4)	0.7382 (3)	0.0599 (13)	
O5A	0.4161 (3)	−0.1296 (2)	0.63054 (19)	0.0336 (8)	
O6A	0.4062 (3)	0.1582 (3)	0.8736 (2)	0.0381 (9)	
N1A	0.2344 (3)	0.1872 (3)	0.6256 (2)	0.0296 (9)	
N2A	0.4552 (3)	0.1178 (3)	0.7425 (2)	0.0294 (9)	
C1A	0.5839 (5)	0.4288 (5)	0.5533 (4)	0.0546 (17)	
H1AA	0.652064	0.424680	0.566692	0.082*	
H1AB	0.563027	0.487155	0.567718	0.082*	
H1AC	0.565151	0.423313	0.503881	0.082*	
C2A	0.5402 (4)	0.3538 (4)	0.5864 (4)	0.0427 (14)	
H2AA	0.559064	0.294447	0.570998	0.051*	
H2AB	0.560679	0.357198	0.636359	0.051*	
C3A	0.3870 (4)	0.2986 (3)	0.5886 (3)	0.0308 (11)	
C4A	0.2923 (4)	0.3166 (3)	0.5742 (3)	0.0333 (11)	
H4AA	0.273743	0.371701	0.551227	0.040*	
C5A	0.2205 (4)	0.2630 (3)	0.5896 (3)	0.0307 (11)	
C6A	0.1225 (4)	0.2945 (4)	0.5617 (3)	0.0407 (13)	
H6AA	0.089920	0.304226	0.599082	0.061*	
H6AB	0.089376	0.248593	0.530828	0.061*	
H6AC	0.124397	0.351271	0.536915	0.061*	
C7A	0.1500 (5)	0.1396 (4)	0.6387 (4)	0.0441 (14)	
H7AA	0.114516	0.180905	0.662741	0.053*	
H7AB	0.109194	0.123153	0.594809	0.053*	
C8A	0.1747 (5)	0.0567 (5)	0.6798 (4)	0.0551 (18)	
H8AA	0.117505	0.031666	0.692046	0.066*	
H8AB	0.199421	0.010996	0.652223	0.066*	
C9A	0.2629 (5)	−0.0069 (4)	0.7766 (4)	0.0471 (15)	
H9AA	0.310156	0.006841	0.817306	0.071*	
H9AB	0.287574	−0.051946	0.749208	0.071*	
H9AC	0.206749	−0.030789	0.789955	0.071*	
C10A	0.3544 (5)	−0.2377 (5)	0.5466 (4)	0.0507 (16)	
H10A	0.324122	−0.246308	0.498918	0.076*	
H10B	0.316328	−0.265642	0.575795	0.076*	
H10C	0.416370	−0.265792	0.555085	0.076*	
C11A	0.3641 (5)	−0.1402 (4)	0.5616 (3)	0.0425 (14)	
H11A	0.397559	−0.110298	0.529497	0.051*	
H11B	0.301623	−0.112036	0.556678	0.051*	
C12A	0.4215 (4)	−0.0453 (3)	0.6573 (3)	0.0285 (10)	
C13A	0.4795 (4)	−0.0382 (3)	0.7205 (3)	0.0281 (10)	
H13A	0.509933	−0.091839	0.739392	0.034*	
C14A	0.4976 (3)	0.0410 (3)	0.7595 (3)	0.0261 (10)	
C15A	0.5742 (4)	0.0330 (4)	0.8228 (3)	0.0335 (11)	
H15A	0.552400	0.059895	0.861251	0.050*	
H15B	0.630200	0.064945	0.815761	0.050*	
H15C	0.589160	−0.030845	0.832241	0.050*	
C16A	0.4798 (4)	0.1990 (3)	0.7836 (3)	0.0320 (11)	
H16A	0.489866	0.249702	0.753800	0.038*	
H16B	0.539023	0.188423	0.816527	0.038*	
C17A	0.4043 (4)	0.2243 (4)	0.8214 (3)	0.0338 (11)	
H17A	0.416101	0.285244	0.841526	0.041*	
H17B	0.342492	0.224453	0.790183	0.041*	
C18A	0.3342 (5)	0.1735 (5)	0.9087 (4)	0.0500 (16)	
H18A	0.338404	0.129244	0.945369	0.075*	
H18B	0.273718	0.167358	0.877353	0.075*	
H18C	0.340015	0.234610	0.927785	0.075*	
Zn2	0.85410 (5)	0.09199 (5)	0.66562 (4)	0.0317 (2)	
O1B	0.9268 (3)	0.0149 (3)	0.6119 (2)	0.0359 (9)	
O2B	0.8621 (3)	0.2179 (2)	0.6254 (2)	0.0366 (9)	
O3B	0.9421 (3)	−0.1030 (3)	0.5452 (3)	0.0504 (12)	
O4B	0.7311 (3)	0.1518 (3)	0.7363 (2)	0.0416 (10)	
O5B	0.9141 (3)	0.3605 (3)	0.6238 (2)	0.0409 (10)	
O6B	0.8534 (3)	−0.0450 (3)	0.7393 (2)	0.0410 (9)	
N1B	0.7292 (3)	0.0503 (3)	0.6164 (2)	0.0298 (9)	
N2B	0.9467 (3)	0.1229 (3)	0.7504 (3)	0.0351 (10)	
C1B	1.0900 (6)	−0.1661 (6)	0.5398 (5)	0.058 (2)	0.717 (13)
H1B1	1.064868	−0.225616	0.547854	0.087*	0.717 (13)
H1B2	1.157226	−0.164792	0.559060	0.087*	0.717 (13)
H1B3	1.079517	−0.154666	0.490931	0.087*	0.717 (13)
C2B	1.0416 (7)	−0.0943 (6)	0.5732 (6)	0.054 (2)	0.717 (13)
H2B1	1.054296	−0.103284	0.623011	0.065*	0.717 (13)
H2B2	1.064031	−0.033569	0.563575	0.065*	0.717 (13)
C1D	1.0829 (14)	−0.1511 (10)	0.6037 (8)	0.056 (3)	0.283 (13)
H1D1	1.056637	−0.139070	0.643915	0.084*	0.283 (13)
H1D2	1.150822	−0.142764	0.615077	0.084*	0.283 (13)
H1D3	1.068639	−0.213407	0.588584	0.084*	0.283 (13)
C2D	1.0410 (18)	−0.0867 (11)	0.5477 (8)	0.054 (3)	0.283 (13)
H2D1	1.058486	−0.023274	0.559738	0.065*	0.283 (13)
H2D2	1.059044	−0.102287	0.504327	0.065*	0.283 (13)
C3B	0.8874 (4)	−0.0465 (4)	0.5736 (3)	0.0348 (12)	
C4B	0.7930 (4)	−0.0664 (4)	0.5550 (3)	0.0367 (12)	
H4BA	0.776373	−0.116307	0.525303	0.044*	
C5B	0.7193 (4)	−0.0205 (4)	0.5756 (3)	0.0316 (11)	
C6B	0.6228 (4)	−0.0594 (4)	0.5486 (3)	0.0417 (14)	
H6BA	0.595931	−0.080524	0.586662	0.063*	
H6BB	0.627730	−0.110194	0.518323	0.063*	
H6BC	0.582572	−0.012613	0.523557	0.063*	
C7B	0.6456 (4)	0.0888 (4)	0.6343 (3)	0.0371 (12)	
H7BA	0.617737	0.043994	0.660856	0.044*	
H7BB	0.599232	0.102002	0.592303	0.044*	
C8B	0.6667 (4)	0.1750 (4)	0.6755 (3)	0.0411 (14)	
H8BA	0.694399	0.220785	0.649594	0.049*	
H8BB	0.608736	0.200129	0.686307	0.049*	
C9B	0.7565 (5)	0.2290 (5)	0.7778 (4)	0.0518 (17)	
H9BA	0.800679	0.211271	0.819136	0.078*	
H9BB	0.700500	0.255088	0.789992	0.078*	
H9BC	0.785691	0.273847	0.752968	0.078*	
C10B	0.8568 (10)	0.4667 (6)	0.5372 (7)	0.056 (3)	0.68 (3)
H10G	0.817213	0.502189	0.561370	0.083*	0.68 (3)
H10H	0.833107	0.471962	0.488221	0.083*	0.68 (3)
H10I	0.920972	0.489476	0.548616	0.083*	0.68 (3)
C11B	0.8552 (12)	0.3684 (6)	0.5581 (7)	0.0503 (18)	0.68 (3)
H11E	0.790662	0.349670	0.559684	0.060*	0.68 (3)
H11F	0.878482	0.329484	0.525158	0.060*	0.68 (3)
C10D	0.8820 (18)	0.4511 (9)	0.5240 (10)	0.054 (3)	0.32 (3)
H10D	0.880279	0.502662	0.554221	0.081*	0.32 (3)
H10E	0.836771	0.460627	0.481408	0.081*	0.32 (3)
H10F	0.944796	0.445283	0.514391	0.081*	0.32 (3)
C11D	0.857 (2)	0.3656 (9)	0.5578 (11)	0.051 (2)	0.32 (3)
H11C	0.790534	0.366555	0.560710	0.061*	0.32 (3)
H11D	0.868203	0.312119	0.530835	0.061*	0.32 (3)
C12B	0.9140 (4)	0.2785 (3)	0.6554 (3)	0.0330 (11)	
C13B	0.9754 (4)	0.2754 (4)	0.7185 (3)	0.0349 (12)	
H13B	1.009261	0.328896	0.733501	0.042*	
C14B	0.9910 (3)	0.2003 (4)	0.7615 (3)	0.0332 (12)	
C15B	1.0658 (5)	0.2141 (5)	0.8255 (4)	0.0558 (18)	
H15D	1.111591	0.165104	0.829503	0.084*	
H15E	1.036701	0.213985	0.865293	0.084*	
H15F	1.097111	0.272146	0.822763	0.084*	
C16B	0.9706 (5)	0.0497 (4)	0.7992 (4)	0.0479 (16)	
H16C	0.998356	0.075424	0.844182	0.058*	
H16D	1.018013	0.010487	0.785051	0.058*	
C17B	0.8880 (5)	−0.0057 (4)	0.8052 (3)	0.0461 (15)	
H17C	0.906039	−0.053657	0.839618	0.055*	
H17D	0.839432	0.032532	0.818823	0.055*	
C18B	0.7731 (5)	−0.1005 (5)	0.7379 (4)	0.0502 (16)	
H18D	0.754932	−0.128955	0.693186	0.075*	
H18E	0.721432	−0.063302	0.746750	0.075*	
H18F	0.787777	−0.147470	0.772798	0.075*	

### Atomic displacement parameters (Å^2^)

**Table:**

	*U*^11^	*U*^22^	*U*^33^	*U*^12^	*U*^13^	*U*^23^
Zn1	0.0219 (4)	0.0181 (3)	0.0519 (4)	0.0018 (2)	0.0047 (3)	0.0036 (3)
O1A	0.0216 (19)	0.0256 (18)	0.061 (2)	0.0044 (15)	0.0111 (16)	0.0087 (17)
O2A	0.038 (2)	0.0224 (18)	0.052 (2)	0.0057 (16)	−0.0012 (17)	−0.0002 (16)
O3A	0.029 (2)	0.0264 (19)	0.058 (2)	0.0023 (15)	0.0106 (17)	0.0113 (16)
O4A	0.053 (3)	0.051 (3)	0.071 (3)	−0.011 (2)	0.001 (2)	0.012 (2)
O5A	0.034 (2)	0.0217 (17)	0.044 (2)	0.0024 (15)	0.0055 (16)	−0.0039 (15)
O6A	0.031 (2)	0.037 (2)	0.049 (2)	−0.0001 (17)	0.0138 (17)	−0.0019 (17)
N1A	0.021 (2)	0.022 (2)	0.046 (2)	0.0006 (16)	0.0078 (17)	−0.0042 (17)
N2A	0.030 (2)	0.0163 (18)	0.043 (2)	−0.0025 (17)	0.0109 (18)	−0.0009 (17)
C1A	0.046 (4)	0.038 (3)	0.086 (5)	0.002 (3)	0.026 (3)	0.007 (3)
C2A	0.029 (3)	0.036 (3)	0.065 (4)	0.001 (2)	0.015 (3)	0.003 (3)
C3A	0.027 (3)	0.020 (2)	0.046 (3)	0.000 (2)	0.010 (2)	−0.001 (2)
C4A	0.031 (3)	0.022 (2)	0.048 (3)	0.006 (2)	0.011 (2)	0.006 (2)
C5A	0.024 (3)	0.024 (2)	0.043 (3)	0.004 (2)	0.005 (2)	−0.008 (2)
C6A	0.030 (3)	0.034 (3)	0.056 (3)	0.010 (2)	0.003 (2)	0.000 (2)
C7A	0.033 (3)	0.035 (3)	0.062 (4)	−0.005 (2)	0.003 (3)	0.002 (3)
C8A	0.052 (4)	0.057 (4)	0.054 (4)	−0.019 (3)	0.003 (3)	0.009 (3)
C9A	0.036 (3)	0.037 (3)	0.070 (4)	−0.004 (3)	0.016 (3)	0.010 (3)
C10A	0.053 (4)	0.041 (3)	0.055 (4)	0.005 (3)	0.002 (3)	−0.009 (3)
C11A	0.042 (3)	0.030 (3)	0.050 (3)	−0.001 (2)	−0.004 (3)	−0.003 (2)
C12A	0.022 (2)	0.016 (2)	0.049 (3)	0.0020 (18)	0.011 (2)	0.001 (2)
C13A	0.025 (3)	0.015 (2)	0.046 (3)	0.0032 (18)	0.012 (2)	0.0032 (19)
C14A	0.013 (2)	0.022 (2)	0.044 (3)	−0.0019 (17)	0.0064 (18)	0.0022 (19)
C15A	0.027 (3)	0.027 (3)	0.046 (3)	−0.002 (2)	0.007 (2)	0.000 (2)
C16A	0.028 (3)	0.019 (2)	0.049 (3)	−0.003 (2)	0.007 (2)	−0.005 (2)
C17A	0.028 (3)	0.024 (2)	0.050 (3)	0.001 (2)	0.010 (2)	−0.004 (2)
C18A	0.041 (4)	0.057 (4)	0.058 (4)	0.007 (3)	0.023 (3)	−0.006 (3)
Zn2	0.0195 (4)	0.0219 (4)	0.0528 (4)	−0.0002 (3)	0.0056 (3)	−0.0014 (3)
O1B	0.0205 (19)	0.0286 (19)	0.059 (2)	−0.0041 (15)	0.0090 (16)	−0.0085 (17)
O2B	0.031 (2)	0.0206 (17)	0.058 (2)	−0.0056 (15)	0.0085 (17)	0.0033 (16)
O3B	0.025 (2)	0.042 (2)	0.086 (3)	−0.0044 (18)	0.014 (2)	−0.027 (2)
O4B	0.030 (2)	0.033 (2)	0.061 (2)	0.0036 (17)	0.0077 (18)	0.0066 (18)
O5B	0.026 (2)	0.0266 (19)	0.070 (3)	−0.0044 (16)	0.0094 (18)	0.0091 (18)
O6B	0.030 (2)	0.032 (2)	0.061 (2)	−0.0020 (17)	0.0095 (18)	0.0099 (18)
N1B	0.0108 (19)	0.032 (2)	0.047 (2)	−0.0010 (16)	0.0071 (16)	0.0105 (19)
N2B	0.024 (2)	0.026 (2)	0.053 (3)	0.0025 (18)	0.0008 (19)	0.0023 (19)
C1B	0.027 (4)	0.055 (4)	0.097 (6)	−0.003 (3)	0.024 (4)	−0.024 (4)
C2B	0.022 (3)	0.046 (4)	0.096 (6)	−0.004 (3)	0.019 (4)	−0.021 (4)
C1D	0.023 (5)	0.048 (6)	0.098 (7)	−0.009 (5)	0.017 (6)	−0.016 (5)
C2D	0.022 (4)	0.047 (5)	0.097 (6)	−0.006 (4)	0.020 (5)	−0.018 (5)
C3B	0.020 (3)	0.030 (3)	0.057 (3)	−0.002 (2)	0.014 (2)	−0.005 (2)
C4B	0.024 (3)	0.030 (3)	0.056 (3)	−0.005 (2)	0.008 (2)	0.000 (2)
C5B	0.022 (3)	0.029 (3)	0.044 (3)	−0.003 (2)	0.007 (2)	0.011 (2)
C6B	0.021 (3)	0.040 (3)	0.063 (4)	−0.005 (2)	0.004 (2)	0.017 (3)
C7B	0.021 (3)	0.039 (3)	0.053 (3)	0.006 (2)	0.011 (2)	0.014 (2)
C8B	0.026 (3)	0.036 (3)	0.065 (4)	0.013 (2)	0.018 (2)	0.019 (3)
C9B	0.035 (3)	0.045 (4)	0.079 (5)	0.004 (3)	0.020 (3)	−0.011 (3)
C10B	0.048 (5)	0.037 (4)	0.078 (5)	0.002 (4)	0.005 (4)	0.015 (4)
C11B	0.040 (4)	0.036 (3)	0.070 (4)	0.000 (3)	0.002 (3)	0.011 (3)
C10D	0.045 (6)	0.038 (5)	0.075 (6)	−0.001 (5)	0.003 (6)	0.012 (5)
C11D	0.042 (4)	0.037 (4)	0.071 (5)	0.000 (4)	0.002 (4)	0.011 (4)
C12B	0.019 (2)	0.021 (2)	0.063 (3)	0.0028 (19)	0.017 (2)	0.003 (2)
C13B	0.017 (2)	0.028 (3)	0.061 (3)	−0.006 (2)	0.012 (2)	−0.003 (2)
C14B	0.009 (2)	0.034 (3)	0.055 (3)	−0.0024 (19)	0.004 (2)	−0.003 (2)
C15B	0.043 (4)	0.049 (4)	0.068 (4)	−0.013 (3)	−0.008 (3)	0.005 (3)
C16B	0.038 (3)	0.036 (3)	0.062 (4)	0.001 (3)	−0.007 (3)	0.010 (3)
C17B	0.047 (4)	0.034 (3)	0.055 (3)	0.007 (3)	0.005 (3)	0.014 (3)
C18B	0.039 (4)	0.043 (3)	0.068 (4)	−0.007 (3)	0.008 (3)	0.020 (3)

### Geometric parameters (Å, º)

**Table:**

Zn1—N1A	1.958 (4)	O3B—C3B	1.361 (7)
Zn1—N2A	1.966 (5)	O3B—C2B	1.456 (11)
Zn1—O2A	1.974 (4)	O3B—C2D	1.46 (3)
Zn1—O1A	2.025 (4)	O4B—C9B	1.419 (8)
Zn1—O4A	2.727 (6)	O4B—C8B	1.425 (7)
O1A—C3A	1.246 (6)	O5B—C12B	1.371 (7)
O2A—C12A	1.271 (6)	O5B—C11D	1.42 (3)
O3A—C3A	1.364 (6)	O5B—C11B	1.429 (15)
O3A—C2A	1.444 (7)	O6B—C18B	1.429 (7)
O4A—C8A	1.376 (8)	O6B—C17B	1.442 (8)
O4A—C9A	1.412 (8)	N1B—C5B	1.322 (7)
O5A—C12A	1.355 (6)	N1B—C7B	1.458 (7)
O5A—C11A	1.450 (7)	N2B—C14B	1.312 (7)
O6A—C18A	1.400 (7)	N2B—C16B	1.457 (8)
O6A—C17A	1.431 (7)	C1B—C2B	1.509 (10)
N1A—C5A	1.329 (7)	C1B—H1B1	0.9800
N1A—C7A	1.490 (8)	C1B—H1B2	0.9800
N2A—C14A	1.306 (7)	C1B—H1B3	0.9800
N2A—C16A	1.462 (6)	C2B—H2B1	0.9900
C1A—C2A	1.502 (9)	C2B—H2B2	0.9900
C1A—H1AA	0.9800	C1D—C2D	1.508 (11)
C1A—H1AB	0.9800	C1D—H1D1	0.9800
C1A—H1AC	0.9800	C1D—H1D2	0.9800
C2A—H2AA	0.9900	C1D—H1D3	0.9800
C2A—H2AB	0.9900	C2D—H2D1	0.9900
C3A—C4A	1.382 (8)	C2D—H2D2	0.9900
C4A—C5A	1.399 (8)	C3B—C4B	1.387 (8)
C4A—H4AA	0.9500	C4B—C5B	1.405 (8)
C5A—C6A	1.502 (7)	C4B—H4BA	0.9500
C6A—H6AA	0.9800	C5B—C6B	1.518 (7)
C6A—H6AB	0.9800	C6B—H6BA	0.9800
C6A—H6AC	0.9800	C6B—H6BB	0.9800
C7A—C8A	1.483 (9)	C6B—H6BC	0.9800
C7A—H7AA	0.9900	C7B—C8B	1.518 (9)
C7A—H7AB	0.9900	C7B—H7BA	0.9900
C8A—H8AA	0.9900	C7B—H7BB	0.9900
C8A—H8AB	0.9900	C8B—H8BA	0.9900
C9A—H9AA	0.9800	C8B—H8BB	0.9900
C9A—H9AB	0.9800	C9B—H9BA	0.9800
C9A—H9AC	0.9800	C9B—H9BB	0.9800
C10A—C11A	1.475 (9)	C9B—H9BC	0.9800
C10A—H10A	0.9800	C10B—C11B	1.515 (9)
C10A—H10B	0.9800	C10B—H10G	0.9800
C10A—H10C	0.9800	C10B—H10H	0.9800
C11A—H11A	0.9900	C10B—H10I	0.9800
C11A—H11B	0.9900	C11B—H11E	0.9900
C12A—C13A	1.384 (8)	C11B—H11F	0.9900
C13A—C14A	1.406 (7)	C10D—C11D	1.515 (9)
C13A—H13A	0.9500	C10D—H10D	0.9800
C14A—C15A	1.520 (7)	C10D—H10E	0.9800
C15A—H15A	0.9800	C10D—H10F	0.9800
C15A—H15B	0.9800	C11D—H11C	0.9900
C15A—H15C	0.9799	C11D—H11D	0.9900
C16A—C17A	1.511 (8)	C12B—C13B	1.400 (8)
C16A—H16A	0.9900	C13B—C14B	1.398 (8)
C16A—H16B	0.9900	C13B—H13B	0.9500
C17A—H17A	0.9900	C14B—C15B	1.525 (8)
C17A—H17B	0.9900	C15B—H15D	0.9801
C18A—H18A	0.9800	C15B—H15E	0.9800
C18A—H18B	0.9800	C15B—H15F	0.9800
C18A—H18C	0.9800	C16B—C17B	1.484 (9)
Zn2—N1B	1.990 (4)	C16B—H16C	0.9900
Zn2—N2B	2.004 (5)	C16B—H16D	0.9900
Zn2—O1B	2.017 (4)	C17B—H17C	0.9900
Zn2—O2B	2.045 (4)	C17B—H17D	0.9900
Zn2—O6B	2.514 (4)	C18B—H18D	0.9800
Zn2—O4B	2.661 (4)	C18B—H18E	0.9800
O1B—C3B	1.253 (7)	C18B—H18F	0.9800
O2B—C12B	1.247 (7)		
			
N1A—Zn1—N2A	144.05 (19)	C3B—O3B—C2B	114.1 (5)
N1A—Zn1—O2A	112.56 (17)	C3B—O3B—C2D	123.2 (8)
N2A—Zn1—O2A	94.98 (17)	C9B—O4B—C8B	111.1 (5)
N1A—Zn1—O1A	95.04 (17)	C9B—O4B—Zn2	117.3 (4)
N2A—Zn1—O1A	96.25 (17)	C8B—O4B—Zn2	91.2 (3)
O2A—Zn1—O1A	110.72 (18)	C12B—O5B—C11D	115.2 (7)
N1A—Zn1—O4A	71.83 (18)	C12B—O5B—C11B	116.4 (5)
N2A—Zn1—O4A	84.34 (17)	C18B—O6B—C17B	112.6 (5)
O2A—Zn1—O4A	93.49 (18)	C18B—O6B—Zn2	123.6 (4)
O1A—Zn1—O4A	155.58 (17)	C17B—O6B—Zn2	100.0 (3)
C3A—O1A—Zn1	121.6 (3)	C5B—N1B—C7B	118.2 (5)
C12A—O2A—Zn1	120.7 (4)	C5B—N1B—Zn2	121.9 (4)
C3A—O3A—C2A	116.8 (4)	C7B—N1B—Zn2	119.1 (4)
C8A—O4A—C9A	111.7 (6)	C14B—N2B—C16B	119.5 (5)
C8A—O4A—Zn1	88.8 (4)	C14B—N2B—Zn2	124.7 (4)
C9A—O4A—Zn1	119.6 (4)	C16B—N2B—Zn2	115.6 (4)
C12A—O5A—C11A	117.2 (4)	C2B—C1B—H1B1	109.5
C18A—O6A—C17A	110.8 (5)	C2B—C1B—H1B2	109.5
C5A—N1A—C7A	117.1 (5)	H1B1—C1B—H1B2	109.5
C5A—N1A—Zn1	123.3 (4)	C2B—C1B—H1B3	109.5
C7A—N1A—Zn1	119.6 (4)	H1B1—C1B—H1B3	109.5
C14A—N2A—C16A	121.2 (5)	H1B2—C1B—H1B3	109.5
C14A—N2A—Zn1	124.2 (4)	O3B—C2B—C1B	106.7 (7)
C16A—N2A—Zn1	114.6 (3)	O3B—C2B—H2B1	110.4
C2A—C1A—H1AA	109.5	C1B—C2B—H2B1	110.4
C2A—C1A—H1AB	109.5	O3B—C2B—H2B2	110.4
H1AA—C1A—H1AB	109.5	C1B—C2B—H2B2	110.4
C2A—C1A—H1AC	109.5	H2B1—C2B—H2B2	108.6
H1AA—C1A—H1AC	109.5	C2D—C1D—H1D1	109.5
H1AB—C1A—H1AC	109.5	C2D—C1D—H1D2	109.5
O3A—C2A—C1A	107.7 (5)	H1D1—C1D—H1D2	109.5
O3A—C2A—H2AA	110.2	C2D—C1D—H1D3	109.5
C1A—C2A—H2AA	110.2	H1D1—C1D—H1D3	109.5
O3A—C2A—H2AB	110.2	H1D2—C1D—H1D3	109.5
C1A—C2A—H2AB	110.2	O3B—C2D—C1D	99.9 (15)
H2AA—C2A—H2AB	108.5	O3B—C2D—H2D1	111.8
O1A—C3A—O3A	118.0 (5)	C1D—C2D—H2D1	111.8
O1A—C3A—C4A	128.0 (5)	O3B—C2D—H2D2	111.8
O3A—C3A—C4A	114.0 (5)	C1D—C2D—H2D2	111.8
C3A—C4A—C5A	127.6 (5)	H2D1—C2D—H2D2	109.5
C3A—C4A—H4AA	116.2	O1B—C3B—O3B	117.9 (5)
C5A—C4A—H4AA	116.2	O1B—C3B—C4B	128.9 (5)
N1A—C5A—C4A	124.1 (5)	O3B—C3B—C4B	113.2 (5)
N1A—C5A—C6A	119.7 (5)	C3B—C4B—C5B	126.8 (5)
C4A—C5A—C6A	116.3 (5)	C3B—C4B—H4BA	116.6
C5A—C6A—H6AA	109.5	C5B—C4B—H4BA	116.6
C5A—C6A—H6AB	109.5	N1B—C5B—C4B	125.0 (5)
H6AA—C6A—H6AB	109.5	N1B—C5B—C6B	120.0 (5)
C5A—C6A—H6AC	109.5	C4B—C5B—C6B	115.1 (5)
H6AA—C6A—H6AC	109.5	C5B—C6B—H6BA	109.5
H6AB—C6A—H6AC	109.5	C5B—C6B—H6BB	109.5
C8A—C7A—N1A	111.9 (5)	H6BA—C6B—H6BB	109.5
C8A—C7A—H7AA	109.2	C5B—C6B—H6BC	109.5
N1A—C7A—H7AA	109.2	H6BA—C6B—H6BC	109.5
C8A—C7A—H7AB	109.2	H6BB—C6B—H6BC	109.5
N1A—C7A—H7AB	109.2	N1B—C7B—C8B	112.0 (5)
H7AA—C7A—H7AB	107.9	N1B—C7B—H7BA	109.2
O4A—C8A—C7A	112.5 (6)	C8B—C7B—H7BA	109.2
O4A—C8A—H8AA	109.1	N1B—C7B—H7BB	109.2
C7A—C8A—H8AA	109.1	C8B—C7B—H7BB	109.2
O4A—C8A—H8AB	109.1	H7BA—C7B—H7BB	107.9
C7A—C8A—H8AB	109.1	O4B—C8B—C7B	107.0 (4)
H8AA—C8A—H8AB	107.8	O4B—C8B—H8BA	110.3
O4A—C9A—H9AA	109.5	C7B—C8B—H8BA	110.3
O4A—C9A—H9AB	109.5	O4B—C8B—H8BB	110.3
H9AA—C9A—H9AB	109.5	C7B—C8B—H8BB	110.3
O4A—C9A—H9AC	109.5	H8BA—C8B—H8BB	108.6
H9AA—C9A—H9AC	109.5	O4B—C9B—H9BA	109.5
H9AB—C9A—H9AC	109.5	O4B—C9B—H9BB	109.5
C11A—C10A—H10A	109.5	H9BA—C9B—H9BB	109.5
C11A—C10A—H10B	109.5	O4B—C9B—H9BC	109.5
H10A—C10A—H10B	109.5	H9BA—C9B—H9BC	109.5
C11A—C10A—H10C	109.5	H9BB—C9B—H9BC	109.5
H10A—C10A—H10C	109.5	C11B—C10B—H10G	109.5
H10B—C10A—H10C	109.5	C11B—C10B—H10H	109.5
O5A—C11A—C10A	108.1 (5)	H10G—C10B—H10H	109.5
O5A—C11A—H11A	110.1	C11B—C10B—H10I	109.5
C10A—C11A—H11A	110.1	H10G—C10B—H10I	109.5
O5A—C11A—H11B	110.1	H10H—C10B—H10I	109.5
C10A—C11A—H11B	110.1	O5B—C11B—C10B	107.2 (9)
H11A—C11A—H11B	108.4	O5B—C11B—H11E	110.3
O2A—C12A—O5A	116.8 (5)	C10B—C11B—H11E	110.3
O2A—C12A—C13A	129.0 (5)	O5B—C11B—H11F	110.3
O5A—C12A—C13A	114.2 (4)	C10B—C11B—H11F	110.3
C12A—C13A—C14A	125.9 (5)	H11E—C11B—H11F	108.5
C12A—C13A—H13A	117.0	C11D—C10D—H10D	109.5
C14A—C13A—H13A	117.0	C11D—C10D—H10E	109.5
N2A—C14A—C13A	123.6 (5)	H10D—C10D—H10E	109.5
N2A—C14A—C15A	121.2 (5)	C11D—C10D—H10F	109.5
C13A—C14A—C15A	115.2 (4)	H10D—C10D—H10F	109.5
C14A—C15A—H15A	109.2	H10E—C10D—H10F	109.5
C14A—C15A—H15B	109.6	O5B—C11D—C10D	108.5 (17)
H15A—C15A—H15B	109.5	O5B—C11D—H11C	110.0
C14A—C15A—H15C	109.6	C10D—C11D—H11C	110.0
H15A—C15A—H15C	109.5	O5B—C11D—H11D	110.0
H15B—C15A—H15C	109.5	C10D—C11D—H11D	110.0
N2A—C16A—C17A	111.6 (4)	H11C—C11D—H11D	108.4
N2A—C16A—H16A	109.3	O2B—C12B—O5B	118.0 (5)
C17A—C16A—H16A	109.3	O2B—C12B—C13B	129.1 (5)
N2A—C16A—H16B	109.3	O5B—C12B—C13B	112.9 (5)
C17A—C16A—H16B	109.3	C14B—C13B—C12B	125.5 (5)
H16A—C16A—H16B	108.0	C14B—C13B—H13B	117.2
O6A—C17A—C16A	107.0 (4)	C12B—C13B—H13B	117.2
O6A—C17A—H17A	110.3	N2B—C14B—C13B	125.2 (5)
C16A—C17A—H17A	110.3	N2B—C14B—C15B	120.4 (5)
O6A—C17A—H17B	110.3	C13B—C14B—C15B	114.5 (5)
C16A—C17A—H17B	110.3	C14B—C15B—H15D	109.3
H17A—C17A—H17B	108.6	C14B—C15B—H15E	109.6
O6A—C18A—H18A	109.5	H15D—C15B—H15E	109.5
O6A—C18A—H18B	109.5	C14B—C15B—H15F	109.5
H18A—C18A—H18B	109.5	H15D—C15B—H15F	109.5
O6A—C18A—H18C	109.5	H15E—C15B—H15F	109.5
H18A—C18A—H18C	109.5	N2B—C16B—C17B	112.2 (5)
H18B—C18A—H18C	109.5	N2B—C16B—H16C	109.2
N1B—Zn2—N2B	152.5 (2)	C17B—C16B—H16C	109.2
N1B—Zn2—O1B	95.08 (17)	N2B—C16B—H16D	109.2
N2B—Zn2—O1B	104.33 (18)	C17B—C16B—H16D	109.2
N1B—Zn2—O2B	101.90 (17)	H16C—C16B—H16D	107.9
N2B—Zn2—O2B	92.60 (18)	O6B—C17B—C16B	106.8 (5)
O1B—Zn2—O2B	102.98 (17)	O6B—C17B—H17C	110.4
N1B—Zn2—O6B	86.02 (16)	C16B—C17B—H17C	110.4
N2B—Zn2—O6B	76.39 (17)	O6B—C17B—H17D	110.4
O1B—Zn2—O6B	85.76 (16)	C16B—C17B—H17D	110.4
O2B—Zn2—O6B	167.48 (16)	H17C—C17B—H17D	108.6
N1B—Zn2—O4B	73.58 (16)	O6B—C18B—H18D	109.5
N2B—Zn2—O4B	83.20 (17)	O6B—C18B—H18E	109.5
O1B—Zn2—O4B	164.14 (14)	H18D—C18B—H18E	109.5
O2B—Zn2—O4B	90.42 (15)	O6B—C18B—H18F	109.5
O6B—Zn2—O4B	82.46 (14)	H18D—C18B—H18F	109.5
C3B—O1B—Zn2	120.9 (3)	H18E—C18B—H18F	109.5
C12B—O2B—Zn2	122.8 (4)		
			
C3A—O3A—C2A—C1A	175.4 (5)	C2B—O3B—C3B—O1B	10.7 (9)
Zn1—O1A—C3A—O3A	173.9 (4)	C2D—O3B—C3B—O1B	−10.6 (11)
Zn1—O1A—C3A—C4A	−5.1 (8)	C2B—O3B—C3B—C4B	−169.3 (7)
C2A—O3A—C3A—O1A	−4.7 (8)	C2D—O3B—C3B—C4B	169.4 (8)
C2A—O3A—C3A—C4A	174.4 (5)	O1B—C3B—C4B—C5B	0.3 (11)
O1A—C3A—C4A—C5A	−0.6 (10)	O3B—C3B—C4B—C5B	−179.7 (6)
O3A—C3A—C4A—C5A	−179.6 (5)	C7B—N1B—C5B—C4B	−178.4 (5)
C7A—N1A—C5A—C4A	178.6 (5)	Zn2—N1B—C5B—C4B	−8.9 (7)
Zn1—N1A—C5A—C4A	−2.2 (7)	C7B—N1B—C5B—C6B	0.1 (7)
C7A—N1A—C5A—C6A	−2.7 (7)	Zn2—N1B—C5B—C6B	169.6 (4)
Zn1—N1A—C5A—C6A	176.5 (4)	C3B—C4B—C5B—N1B	0.2 (10)
C3A—C4A—C5A—N1A	4.8 (9)	C3B—C4B—C5B—C6B	−178.3 (6)
C3A—C4A—C5A—C6A	−173.9 (6)	C5B—N1B—C7B—C8B	−174.2 (5)
C5A—N1A—C7A—C8A	−179.3 (5)	Zn2—N1B—C7B—C8B	15.9 (6)
Zn1—N1A—C7A—C8A	1.4 (7)	C9B—O4B—C8B—C7B	179.0 (5)
C9A—O4A—C8A—C7A	−179.8 (6)	Zn2—O4B—C8B—C7B	59.0 (4)
Zn1—O4A—C8A—C7A	−58.2 (6)	N1B—C7B—C8B—O4B	−60.8 (6)
N1A—C7A—C8A—O4A	51.8 (9)	C12B—O5B—C11B—C10B	172.8 (9)
C12A—O5A—C11A—C10A	−171.2 (5)	C12B—O5B—C11D—C10D	−167.1 (14)
Zn1—O2A—C12A—O5A	169.2 (3)	Zn2—O2B—C12B—O5B	179.9 (3)
Zn1—O2A—C12A—C13A	−11.9 (8)	Zn2—O2B—C12B—C13B	0.7 (8)
C11A—O5A—C12A—O2A	5.1 (7)	C11D—O5B—C12B—O2B	−2.9 (15)
C11A—O5A—C12A—C13A	−173.9 (5)	C11B—O5B—C12B—O2B	−1.0 (10)
O2A—C12A—C13A—C14A	1.1 (9)	C11D—O5B—C12B—C13B	176.4 (14)
O5A—C12A—C13A—C14A	180.0 (5)	C11B—O5B—C12B—C13B	178.3 (9)
C16A—N2A—C14A—C13A	−177.4 (5)	O2B—C12B—C13B—C14B	0.4 (10)
Zn1—N2A—C14A—C13A	0.5 (7)	O5B—C12B—C13B—C14B	−178.8 (5)
C16A—N2A—C14A—C15A	0.1 (8)	C16B—N2B—C14B—C13B	179.4 (6)
Zn1—N2A—C14A—C15A	178.0 (4)	Zn2—N2B—C14B—C13B	5.4 (8)
C12A—C13A—C14A—N2A	5.6 (8)	C16B—N2B—C14B—C15B	−1.1 (9)
C12A—C13A—C14A—C15A	−172.0 (5)	Zn2—N2B—C14B—C15B	−175.2 (5)
C14A—N2A—C16A—C17A	−108.3 (6)	C12B—C13B—C14B—N2B	−3.8 (9)
Zn1—N2A—C16A—C17A	73.6 (5)	C12B—C13B—C14B—C15B	176.7 (6)
C18A—O6A—C17A—C16A	−176.4 (5)	C14B—N2B—C16B—C17B	145.5 (6)
N2A—C16A—C17A—O6A	71.1 (6)	Zn2—N2B—C16B—C17B	−39.9 (7)
C3B—O3B—C2B—C1B	175.8 (7)	C18B—O6B—C17B—C16B	−179.9 (5)
C3B—O3B—C2D—C1D	101.5 (11)	Zn2—O6B—C17B—C16B	−46.9 (5)
Zn2—O1B—C3B—O3B	−172.2 (4)	N2B—C16B—C17B—O6B	61.7 (7)
Zn2—O1B—C3B—C4B	7.7 (9)		

### Hydrogen-bond geometry (Å, º)

**Table:**

*D*—H···*A*	*D*—H	H···*A*	*D*···*A*	*D*—H···*A*
C6*A*—H6*AB*···O3*B*^i^	0.98	2.63	3.568 (8)	161
C15*A*—H15*A*···O6*A*	0.98	2.64	3.396 (7)	134
C15*A*—H15*C*···O3*A*^ii^	0.98	2.65	3.369 (7)	131
C18*A*—H18*A*···O3*A*^iii^	0.98	2.60	3.304 (8)	129
C8*B*—H8*BA*···O2*B*	0.99	2.60	3.279 (7)	126

Symmetry codes: (i) −*x*+1, −*y*, −*z*+1; (ii) −*x*+1, *y*−1/2, −*z*+3/2; (iii) *x*, −*y*+1/2, *z*+1/2.
